# Nuclear receptor HNF4α performs a tumor suppressor function in prostate cancer via its induction of p21-driven cellular senescence

**DOI:** 10.1038/s41388-019-1080-3

**Published:** 2019-11-06

**Authors:** Zhu Wang, Youjia Li, Dinglan Wu, Shan Yu, Yuliang Wang, Franky Leung Chan

**Affiliations:** 1 Department of Urology, People’s Hospital of Longhua, Southern Medical University, Shenzhen, Guangdong, 518109 China; 2 School of Biomedical Sciences, The Chinese University of Hong Kong, Hong Kong, China

**Keywords:** Prostate cancer, Oncogenes

## Abstract

Hepatocyte nuclear factor 4α (HNF4α, NR2A1) is a highly conserved member of the nuclear receptor superfamily. Recent advances reveal that it is a key transcriptional regulator of genes, broadly involved in xenobiotic and drug metabolism and also cancers of gastrointestinal tract. However, the exact functional roles of HNF4α in prostate cancer progression are still not fully understood. In this study, we determined the functional significance of HNF4α in prostate cancer. Our results showed that HNF4α exhibited a reduced expression pattern in clinical prostate cancer tissues, prostate cancer cell lines and xenograft model of castration-relapse prostate cancer. Stable HNF4α knockdown not only could promote cell proliferation and suppress doxorubicin (Dox)-induced cellular senescence in prostate cancer cells, but also confer resistance to paclitaxel treatment and enhance colony formation capacity and in vivo tumorigenicity of prostate cancer cells. On the contrary, ectopic overexpression of HNF4α could significantly inhibit the cell proliferation of prostate cancer cells, induce cell-cycle arrest at G_2_/M phase and trigger the cellular senescence in prostate cancer cells by activation of p21 signal pathway in a p53-independent manner via its direct transactivation of *CDKN1A*. Together, our results show that HNF4α performs a tumor suppressor function in prostate cancer via a mechanism of p21-driven cellular senescence.

## Introduction

Evasion of cellular senescence induced by DNA damage-driven genome instability or activation of oncogenes is considered as one of the hallmarks of cancer. Tumor senescence is an important tumor-suppressing mechanism governing the oncogenic transformation and also advanced malignant development of cancer [1]. This process is mainly regulated by two main tumor suppressor pathways, telomere-dependent p19^ARD^/p53/p21^WAF1/CIP1^ and oxidative stress-induced Erk/p38^MAPK^/p16, both leading to pRb activation and arrest of cell-cycle progression via different downstream mediators [2]. Aberrant expressions or mutations of genes involved in these pathways have been demonstrated in some subsets of primary prostate cancer and also associated with its early relapse [3]. It is well known that besides androgens and genetic familial factors, increasing age is a significant risk factor for prostate cancer [4]. In fact, prostate cancer can be divided into two clinical subtypes, the low-risk cancers (clinically indolent and nonlethal) that commonly diagnosed in significant proportion of elderly patients and the high-risk cancers (clinically aggressive and lethal) that diagnosed in patients at younger age [5]. Cellular senescence or tissue aging is implicated in the low-risk prostate cancers in elderly patients [6].

Hepatocyte nuclear factor 4 alpha (HNF4α, NR2A1, *HNF4A*) is a highly conserved member of the nuclear receptor (NR) superfamily. HNF4α was originally cloned from the rat liver cDNA library [7] and its human paralogue was then isolated from the human adult liver cDNA library using the rat cDNA probe [8]. Although HNF4α has been considered as an orphan NR, few studies suggest that some long-chain fatty acids can bind as endogenous ligands to its ligand-binding domain (LBD) and modulate its transcriptional activity [9–11]. HNF4α exhibits high expression in the adult liver, kidney, intestine, pancreas, and some cancer cell lines derived from these organs [7, 8, 12]. HNF4α is characterized to play an important role in liver differentiation and functions via its control of genes involved broadly in intermediary metabolism, including glucose, bile acid, cholesterol, fatty acid, xenobiotic, and drug metabolism [13, 14], and also in pancreatic β-cells [15]. Transgenic knockout studies indicate that HNF4α is essential for visceral endoderm development [16], differentiation, and functional maintenance of hepatocytes [17–19]. HNF4α is linked to several human diseases including diabetes, hepatitis B viral infection, and cancer. Mutations in *HNF4A* gene are linked to maturity-onset diabetes of the young [15]. Mutation analysis and transgenic knockout studies suggest that HNF4α plays an antiinflammatory role in intestinal epithelium and its gene polymorphisms are associated with inflammatory bowel diseases [20–23].

HNF4α is implicated in cancer growth and development. However, it still remains controversial on its exact roles as either tumor suppressing or oncogenic functions in cancers. Altered expressions of HNF4α isoforms formed by alternative promoter usage and splicing are detected in various adenocarcinomas and their metastatic lesions [24, 25]. Downregulation of HNF4α is described in renal cell carcinoma (RCC) [26], hepatocellular carcinoma (HCC) and cirrhotic tissue, colorectal carcinoma [24, 25], and rodent models of HCC [27, 28]. Ectopic expression of HNF4α can inhibit cell proliferation in rodent embryonal carcinoma cells, immortalized lung endothelial cells, pancreatic β-cells [29, 30], and HEK293 human embryonic kidney cells [31]. Enforced HNF4α expression can also suppress epithelial–mesenchymal transition (EMT) via inhibition of β-catenin as shown in a carcinogen-induced rat model of HCC [28]. Moreover, deletion of HNF4α can promote cell proliferation of hepatocytes in mice [32, 33]. These results seem to suggest that HNF4α may perform a tumor suppressive function in RCC and HCC.

On the other hand, HNF4α also shows increased expression in clinical samples of HCC [34], ovarian mucinous carcinomas [35], colorectal carcinoma [36], lung mucinous adenocarcinoma [37], and neuroblastoma [38]. It is shown that HNF4α does not act as a tumor suppressor but can promote intestinal tumorigenesis in the *APC*^*Min*^ mouse model of intestinal carcinoma via its direct regulation of oxidoreductase-related genes and reactive oxygen species production [36]. Overexpression of HNF4α can enhance the aggressiveness and angiogenesis of neuroblastoma cells via its direct upregulation of matrix metalloproteinase 14 (MMP-14) [38]. These conflicting reports implicate that HNF4α may perform different roles in different cancer types or stages of cancer development.

In this study, we characterized the functional significance of HNF4α in the growth regulation of prostate cancer. We showed that HNF4α, which exhibited a downregulation expression in prostate cancer, could suppress the malignant growth of prostate cancer cells via its direct transcriptional regulation of senescence-regulatory gene *CDKN1A* (p21^WAF1/CIP1^).

## Results

### HNF4α exhibits a decreased expression in prostate cancer

**Fig. 1:**
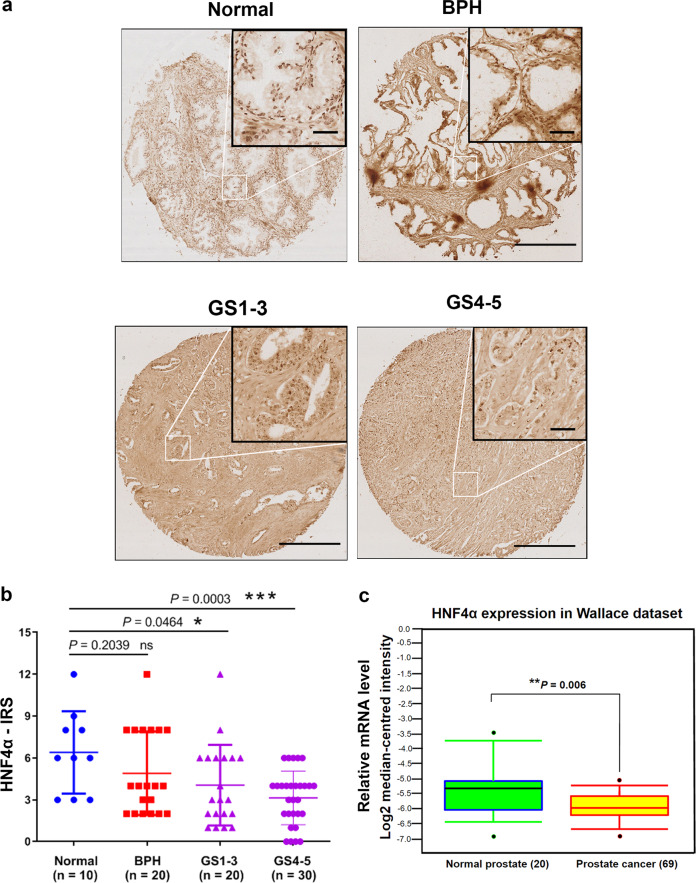
HNF4α exhibits a decreased expression pattern in prostate cancer. **a** Immunohistochemical analysis of HNF4α in tissue microarray. Representative micrographs show the HNF4α-immunostained tissue spots of normal prostate, benign prostatic hyperplasia (BPH), and prostate cancer tissues. The normal prostatic and BPH epithelial cells exhibited moderate immunostaining of HNF4α in their nuclei. The malignant cells in low and moderate differentiated adenocarcinoma lesions (Gleason scores 1–3) showed weak nuclear immunoreactivity and barely detected or negative immunoreactivity in high-grade poorly differentiated lesions (Gleason scores 4–5). Magnification, ×40; scale bars = 500 μm. Insets show the enclosed areas at higher magnification. Magnification ×400; scale bars = 100 µm. **b** HNF4α-IRS analysis performed on malignant and nonmalignant (normal and BPH) prostatic tissues. Results showed that adenocarcinoma lesions exhibited significant lower HNF4α expression than that normal and BPH tissues. *P < 0.05, ***P < 0.001. IRS, immunoreactivity score. **c** Expression profile of HNF4α as revealed in an Oncomine dataset (GSE6956). Results showed that HNF4α mRNA levels exhibited a significant decrease in prostate cancer tissues as compared with normal prostate gland. Box plot (lines from top to bottom): maximum, third quartile Q3; median; minimum, first quartile Q1. **P < 0.01 versus normal prostate

Real-time qRT-PCR and immunoblot analyses of HNF4α expression performed in a panel of immortalized nonmalignant prostatic epithelial and prostate cancer cell lines revealed that HNF4α exhibited a significant decreased expression in all tested prostate cancer cell lines as compared with immortalized prostatic epithelial cell lines (Supplementary Fig. S1a). Similarly, a decreased expression of HNF4α was also observed in two in vitro models of metastatic and androgen-independent prostate cancer, C4-2B [39] and PC-3M [40], as compared with their parental lines LNCaP and PC-3 (Supplementary Fig. S1b). Expression analysis of HNF4α in a castration-resistant prostate cancer (CRPC) xenograft model VCaP-CRPC showed that HNF4α displayed a significant decreased expression in castration-relapse VCaP-CRPC xenograft tumors as compared with precastrated VCaP xenograft tumors (Supplementary Fig. S1c). Immunocytochemical staining also validated that HNF4α exhibited a decrease expression pattern in prostate cancer cells (LNCaP and PC-3) as compare with immortalized epithelial cells PWR-1E and nonprostatic BPH-1 (Supplementary Fig. S2). Immunohistochemistry of HNF4α showed that epithelial cells in normal prostate and benign prostatic hyperplasia (BPH) tissues showed positive nuclear staining. However, malignant cells showed significant reduced nuclear immunoreactivity in high-grade prostatic carcinoma lesions (Fig. 1a). IRS analysis confirmed that high Gleason-scored lesions exhibited significant lower HNF4α immunoreactivity scores as compared with normal or BPH (Fig. 1b). We further analyzed the HNF4α expression profile in clinical prostatic samples using two publicly available gene expression microarray datasets from Oncomine and Gene Expression Omnibus (GSE3868) [41, 42]. Both datasets confirmed that the prostate cancer samples exhibited a lower expression of HNF4α as compared with normal prostate gland or BPH (Fig. 1c and Supplementary Fig. S3). Together, these results showed that HNF4α exhibited a downregulation in prostate cancer and also its advanced progression.

### Downregulation of HNF4α in prostate cancer cells involves epigenetic modifications

**Fig. 2:**
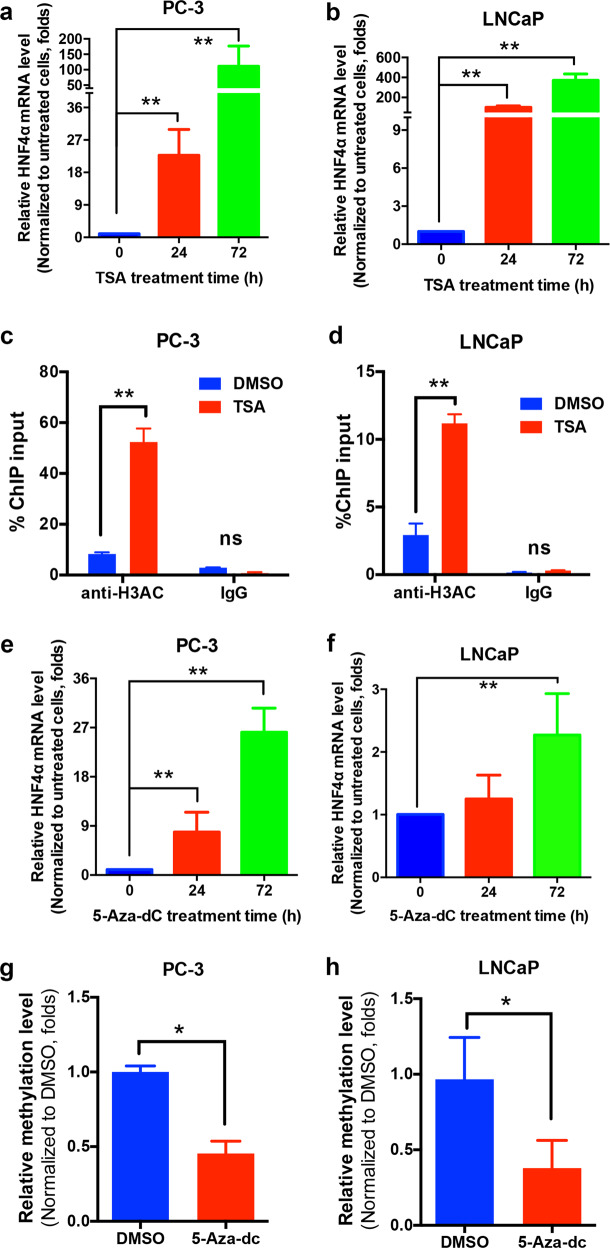
Downregulation of HNF4α in prostate cancer cells involves epigenetic modifications. **a**, **b** qRT-PCR analysis of HNF4α expression. Results showed that HNF4α expression was significantly increased upon treatment with histone deacetylase inhibitor (TSA, 200 ng/ml) in both PC-3 (**a**) and LNCaP (**b**) cells. **c**, **d** ChIP-qPCR assay of *HNF4A* gene using H3Ac antibody (ab47915, abcam). Results validated that acetyl-histone H3 was highly enriched in the *HNF4A* promoter in both PC-3 (**c**) and LNCaP (**d**) cells. **e**, **f** qRT-PCR analysis. Results showed HNF4α expression was rescued in both PC-3 (**e**) and LNCaP (**f**) cells upon treatment with DNA methyltransferase inhibitor (5-aza-dC, 100 nM). **g**, **h** qMSP analysis. Results showed that there was a significant demethylation of the *HNF4A* gene promoter in PC-3 (**g**) and LNCaP (**h**) cells after 5-aza-dC treatment. *P < 0.05, **P < 0.01 versus untreated or DMSO group

We next investigated whether epigenetic events would be involved in the decreased expression of HNF4α in prostate cancer cells. We found that in vitro treatment with a histone deacetylase inhibitor trichostatin A (TSA) could significantly increase the HNF4α expression in an androgen receptor (AR)-negative (PC-3) and two AR-positive (LNCaP, ARCaP_M_) prostate cancer cell lines in a time-dependent manner (Fig. 2a, b; Supplementary Fig. S4a). Chromatin immunoprecipitation (ChIP)-qPCR assay using an anti-acetyl-histone H3 (H3Ac) antibody showed that H3Ac was highly enriched in the HNF4α (*HNF4A*) promoter in PC-3 and LNCaP cells (Fig. 2c, d). Similarly, to clarify whether promoter methylation would also contribute to the HNF4α silencing, we treated prostate cancer cells (PC-3, LNCaP, and ARCaP_M_) with a DNA methyltransferase inhibitor 5-aza-2′-deoxycytidine (5-aza-dC), followed by quantitative methylation-specific PCR (qMSP) analysis. Results showed that HNF4α expression was rescued in prostate cancer cells upon 5-aza-dC treatment, along with demethylation of the *HNF4A* promoter (Fig. 2e–h; Supplementary Fig. S4b). These results indicate that histone deacetylation and DNA methylation could contribute to the epigenetic downregulation of HNF4α expression in prostate cancer cells.

### HNF4α knockdown enhances both in vitro and in vivo malignant growth capacities of prostate cancer cells

**Fig. 3:**
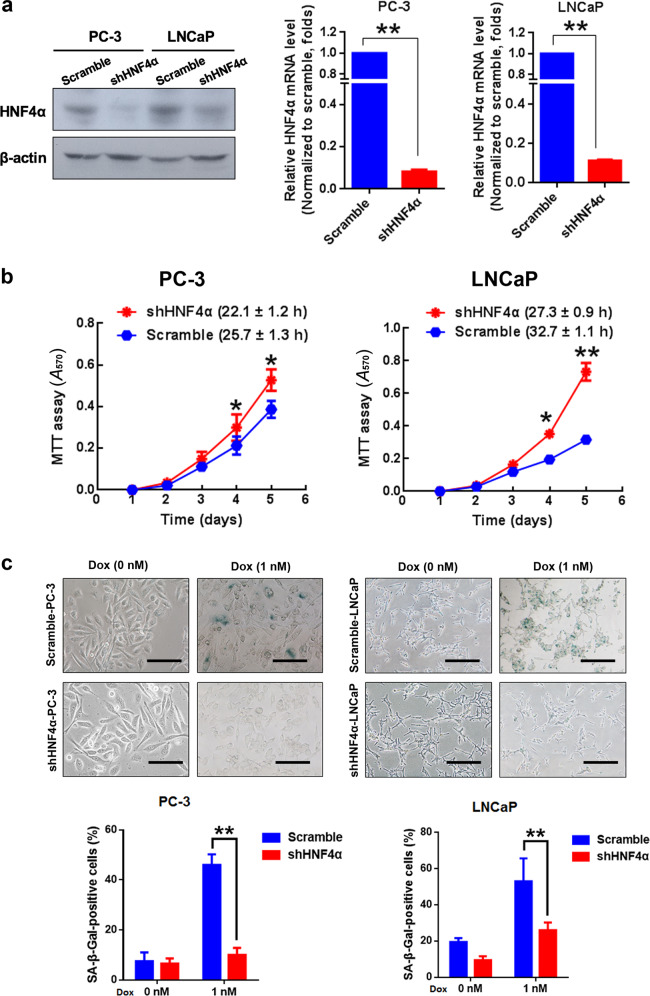
HNF4α knockdown promotes cell proliferation and resistance to Dox-induced cellular senescence in prostate cancer cells. **a** Immunoblot and qRT-PCR validation of shRNA knockdown. Left: immunoblot validation of the constructed HNF4α-specific shRNA expressing vector on its HNF4α-knockdown efficiency in AR-negative PC-3 cells and AR-positive LNCaP cells. Right: qRT-PCR analysis showed that the HNF4α mRNA expression was significantly reduced in shHNF4α-infectants of PC-3 and LNCaP cells. **b** MTT analysis. Both PC-3-shHNF4α and LNCaP-shHNF4α infectants proliferated at faster rates than their shScramble infectants. **c** SA-β-Gal cytochemical assay. Top: micrographs show shHNF4α- and shScramble infectants upon Dox treatment (1 nM). Scale bars = 100 μm. Bottom: graphs show the % of SA-β-Gal-positive cells. Dox treatment induced no significant increase of SA-β-Gal-positive cells in shHNF4α-infectants as compared to shScramble infectants. *P < 0.05, **P < 0.01 versus shScramble infectants

**Fig. 4:**
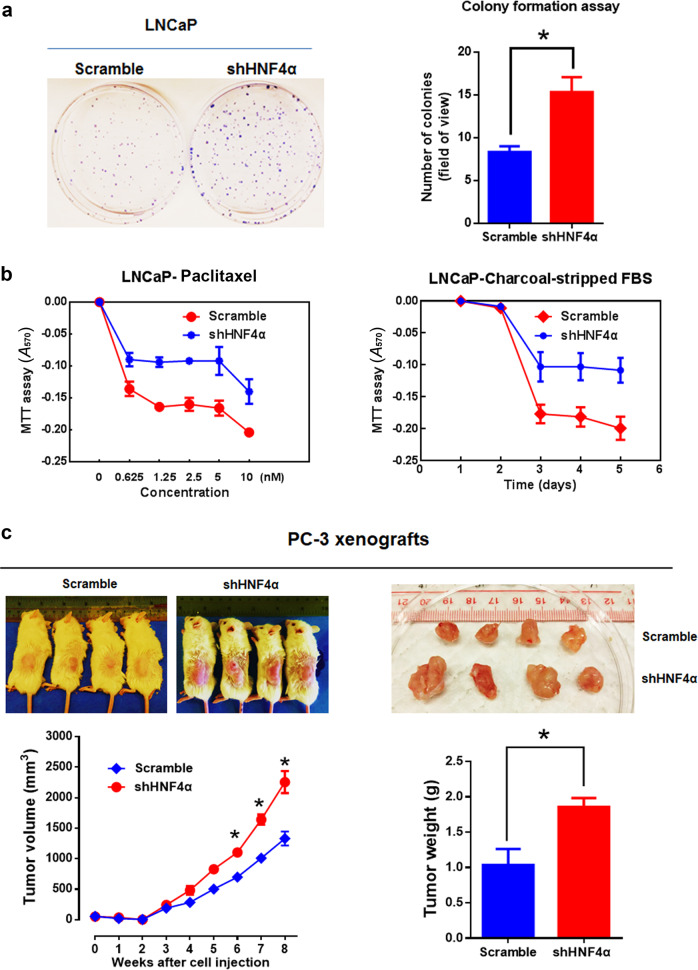
HNF4α knockdown promotes both in vitro and in vivo malignant growth of prostate cancer cells. **a** Clonogenic assay. Left: representative images of stained colonies formed by the LNCaP-shHNF4α and LNCaP-shScramble infectants. Right: histogram shows the colonies formed by the LNCaP-shHNF4α and LNCaP-shScramble infectants. The LNCaP-shHNF4α infectants formed more and larger colonies than the LNCaP-shScramble infectants. **b** MTT assay. The LNCaP-shHNF4α infectants showed higher resistance to paclitaxel (0.625–10 nM) and charcoal-stripped FBS than LNCaP-shScramble infectants. **c** In vivo tumorigenicity assay. Upper panels: images of representative host SCID mice bearing the subcutaneous xenograft tumors formed by the injected PC-3-shHNF4α and PC-3-shScramble infectants (left) and the tumors dissected from the host mice (right). Lower panels: time-growth curve of xenograft tumors formed by the PC-3-shHNF4α and PC-3-shScramble infectants and the histogram of wet weights of tumors dissected from the host mice. The PC-3-shHNF4α infectants formed xenograft tumors at faster rates and with larger sizes than that of PC-3-shScramble infectants. *P < 0.05 versus shScramble infectants

Since HNF4α displayed a decreased expression pattern in prostate cancer tissues and models of prostate cancer, we hypothesize that HNF4α might perform a negative or tumor-suppressing function in prostate cancer. We then generated stable HNF4α-knockdown infectants in two prostate cancer cell lines (LNCaP: AR-positive and wild-type p53-positive; PC-3: AR-negative and p53-deficient) by lentiviral shHNF4α infection (Fig. 3a). In vitro cell proliferation assay showed that the shHNF4α-infectants exhibited faster proliferation rates than their Scramble-infectants (Fig. 3b). Cytochemical analysis of senescence-associated β-galactosidase (SA-β-Gal) activity showed that stable knockdown of HNF4α could confer significant resistance to doxorubicin (Dox)-induced cellular senescence in HNF4α-infectants (Fig. 3c). We also found that knockdown of HNF4α could induce higher colony formation capacity and confer higher resistance to paclitaxel and charcoal-stripped serum (androgen-deprivation) treatments in AR-positive LNCaP-shHNF4α infectants, as compared with shScramble-infectants (Fig. 4a, b). Furthermore, in vivo tumorigenicity analysis of shHNF4α-infectants showed that the PC-3-shHNF4α infectants formed significant larger xenograft tumors in SCID mice as compared with PC-3-shScramble infectants (Fig. 4c). Together, these results showed that knockdown of endogenous HNF4α could promote both in vitro and in vivo malignant growth capacities of prostate cancer cells.

### HNF4α overexpression suppresses both in vitro and in vivo malignant growth of prostate cancer cells

**Fig. 5:**
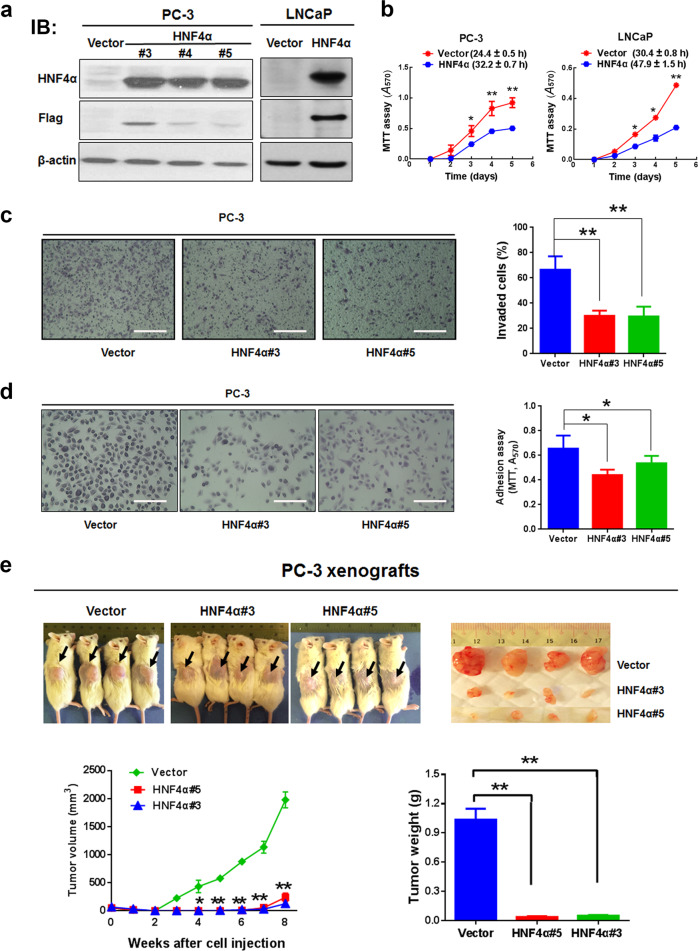
HNF4α overexpression suppresses both in vitro and in vivo malignant growth of prostate cancer cells. **a** Immunoblot validation of HNF4α protein expression in stable HNF4α-infectants generated from PC-3 and LNCaP cells. Vector-infectants were set as controls and detection of Flag-tagged HNF4α was used as positive expression control. **b** Cell viability analysis. The growth curves of HNF4α-overexpressed PC-3 (left) and LNCaP (right) cells were determined by MTT assay. Results showed that HNF4α-infectants grew significantly slower than their corresponding vector-infectants. Brackets show the doubling time (h) of infectants. **c** In vitro invasion assay. Left: representative micrographs show the crystal violet-stained invaded cells on membranes. Scale bars = 100 μm. Right: graph shows the number of invaded cells counted in five randomly selected ×200 fields per transwell insert. All determinations were performed at least in triplicate in three independent experiments. **d** Adhesion assay. Left: representative micrographs show the crystal violet-stained adherent cells. Scale bars = 100 μm. Right: graph shows the adherent cell number as determined by MTT assay. Results showed that there was a significant decrease of adherent cells in HNF4α overexpressed PC-3 cells. All determinations were performed at least in triplicate in three independent experiments. **e** In vivo tumorigenicity assay. Upper: representative images of mice bearing xenograft tumors and dissected tumors formed by HNF4α- or vector-infectants. Lower: time-growth curve of xenograft tumors grown in mice and histogram shows the wet weights of dissected tumors. All determinations were performed at least in triplicate in three independent experiments. The data are presented as the mean ± SD from three independent experiments. *P < 0.05; **P < 0.01 versus vector-infectant controls

To further validate the tumor-suppressing role of HNF4α in prostate cancer cell growth, we next generated stable HNF4α-transduced infectants in AR-negative PC-3 and AR-positive LNCaP cells for growth phenotype characterization. In vitro cell proliferation assay showed that contrary to shHNF4α infectants, all the immunoblot-validated HNF4α-infectants of prostate cancer cells proliferated at slower rates than their empty vector-infectants (Fig. 5a, b). Moreover, both PC-3-HNF4α and LNCaP-HNF4α infectants also exhibited suppressed in vitro invasion and adhesion capacities, as compared with their counterpart empty vector-infectants (Fig. 5c, d). In vivo tumorigenicity assay showed that the HNF4α-infectants grew very small tumors in host mice (Fig. 5e). These results further validated that HNF4α could perform a tumor-suppressing function in prostate cancer cells as its overexpression could repress both in vitro and in vivo malignant growth of prostate cancer cells regardless of their AR and p53 expression status.

### HNF4α overexpression induces cell-cycle arrest and cellular senescence in prostate cancer cells

**Fig. 6:**
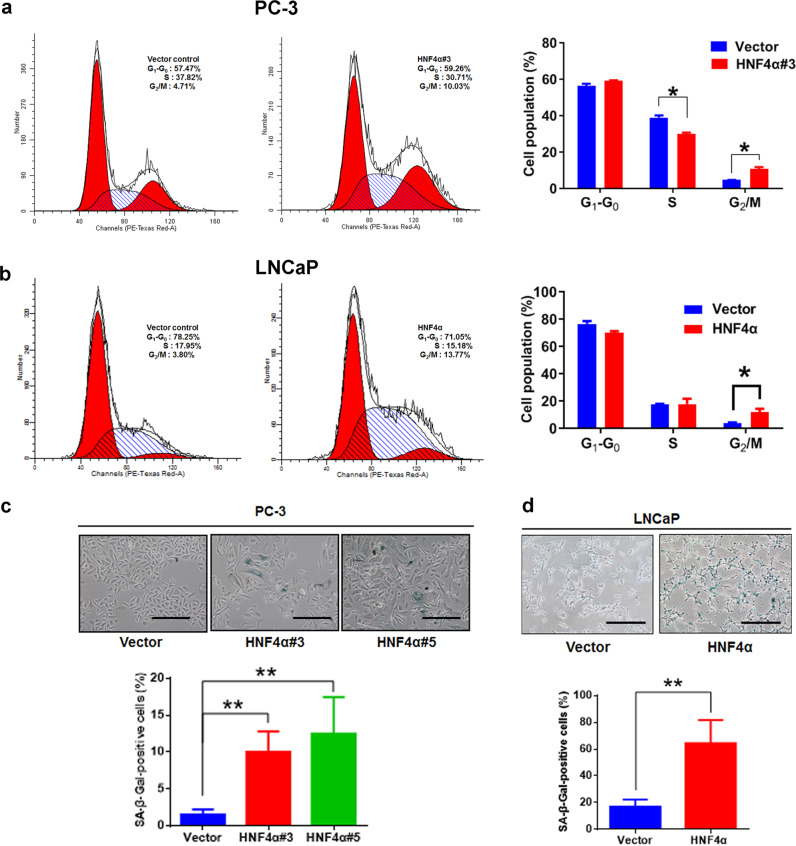
HNF4α overexpression induces cell-cycle arrest and cellular senescence in prostate cancer cells. **a**, **b** Cell cycle analysis. Flow cytometric analysis of PC-3 and LNCaP-HNF4α-infectants and their vector-infectants control shown as DNA histograms. Cell-cycle distribution was represented as the percentage of cells at each phase. Both PC-3 and LNCaP-HNF4α-infectants showed significant higher percentages of cells at G_2_/M phase than their control vector-infectants. **c**, **d** Cytochemical staining of SA-β-Gal activity in HNF4α-infectants. Upper: representative micrographs show the SA-β-Gal staining (perinuclear staining) in HNF4α-infectants. Scale bars = 100 μm. Lower: histograms show the percentages of SA-β-Gal-positive cells in HNF4α-infectants. Results showed that there was a significant increase of SA-β-Gal-positive cells in both PC-3 and LNCaP-HNF4α-infectants as compared with their control vector-infectants. *P < 0.05; **P < 0.01 versus control vector-infectants

To further elucidate the role of HNF4α in the growth suppression of prostate cancer cells, we next analyzed the cell-cycle progression status of HNF4α-infectants by DNA flow cytometry. Results showed that the HNF4α-infectants contained an increased cell population at G_2_M phase, but with no significant presence of apoptotic cells as appeared in sub-G_1_ phase (Fig. 6a, b). Cytochemical analysis of SA-β-Gal activity showed that there were significant increases of SA-β-Gal-positive cells in both PC-3-HNF4α and LNCaP-HNF4α infectants as compared with their corresponding vector-infectants (Fig. 6c, d). Together, these findings showed that overexpression of HNF4α could induce cell-cycle arrest and cellular senescence in prostate cancer cells, resulted in their growth suppression.

### Knockdown of HNF4α expression prevents cellular senescence induced by activated oncogene H-Ras^G12V^ or loss-of-PTEN in prostatic epithelial cells

**Fig. 7:**
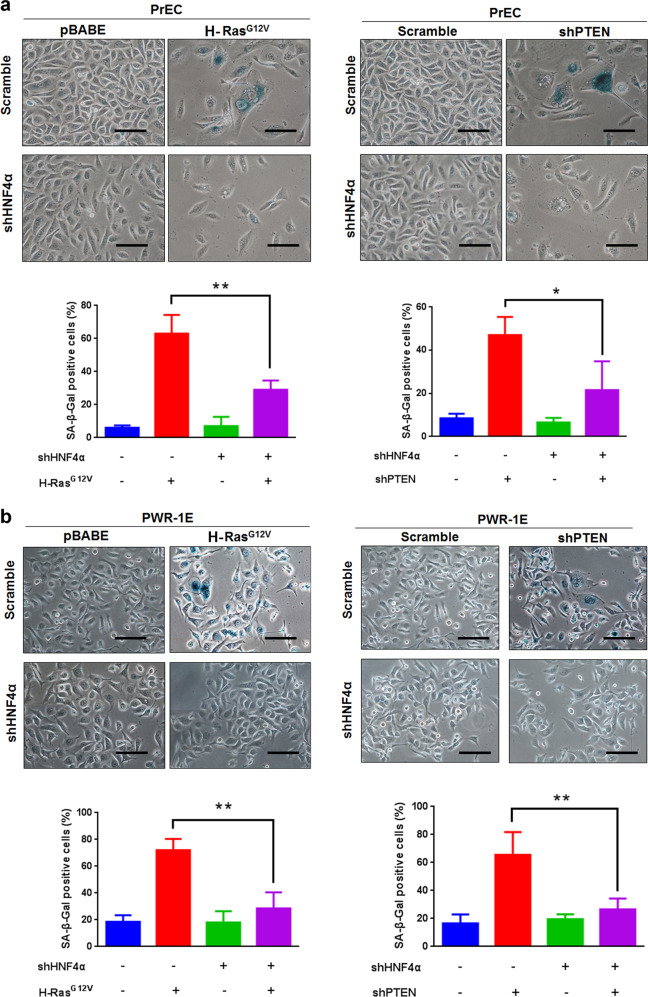
Knockdown of HNF4α prevents cellular senescence induced by activated oncogene H-Ras^G12V^ or loss-of-PTEN in prostatic epithelial cells. Evaluation of SA-β-Gal activity in Ras^G12V^-transduced and shPTEN-transduced (**a**) PrEC and (**b**) PWR-1E cells upon HNF4α knockdown. Upper: representative micrographs show the SA-β-Gal-positive cells detected in Ras^G12V^-transduced (left panels) and shPTEN-transduced (right panels) PrEC and PWR-1E cells with or without HNF4α knockdown. Lower: histograms show the scores of SA-β-Gal-positive cells in Ras^G12V^ and shPTEN-transduced PrEC and PWR-1E cells with or without HNF4α knockdown. Magnification, × 200; scale bars = 100 μm. *P < 0.05; **P < 0.01 versus no shHNF4α infection

To evaluate whether HNF4α could play a critical role in the progression of cellular senescence, we next generated oncogene-induced senescence model in immortalized PWR-1E and primary cultured prostatic epithelial cells by ectopic overexpression of a constitutively activated oncogene, H-Ras^G12V^. Results showed that ectopic expression of oncogenic H-Ras^G12V^ could significantly induce cellular senescence features in both PWR-1E and PrEC cells, as evidenced by their enlarged flattened phenotypes and enhanced SA-β-Gal staining. However, ectopic H-Ras^G12V^ expression could not induce significant cellular senescence in HNF4α-knockdowned PrEC or PWR-1E cells, as shown by their low scores of SA-β-Gal-positive cells (Fig. 7). Since loss or inactivation of tumor suppressor PTEN is well-characterized to trigger cell senescence in mouse prostate [43, 44] and also play a crucial role in the development and advance progression of prostate cancer [45–47], we then examined the possible association between HNF4α and PTEN in prostate cancer cells with HNF4α overexpression. Results of qRT-PCR showed that ectopic HNF4α overexpression induced no significant changes in *PTEN* transcript levels in prostate cancer cells (Supplementary Fig. S5). We next examined the significance of HNF4α in senescence induced by loss of tumor suppressor *PTEN* gene in PrEC and PWR-1E cells. Results showed that HNF4α-knockdown could significantly rescue cellular senescence induced by shRNA-mediated knockdown of *PTEN* in PrEC and PWR-1E cells (Fig. 7). Taking together, these results indicated that HNF4α could play a critical role in prevention of cellular senescence induced by either activated oncogene such as H-Ras^G12V^ or loss of *PTEN* in prostatic cells.

### HNF4α-induced cellular senescence in prostate cancer cells is mediated via its direct transactivation of *CDKN1A* (p21^WAF1/CIP1^) gene

**Fig. 8:**
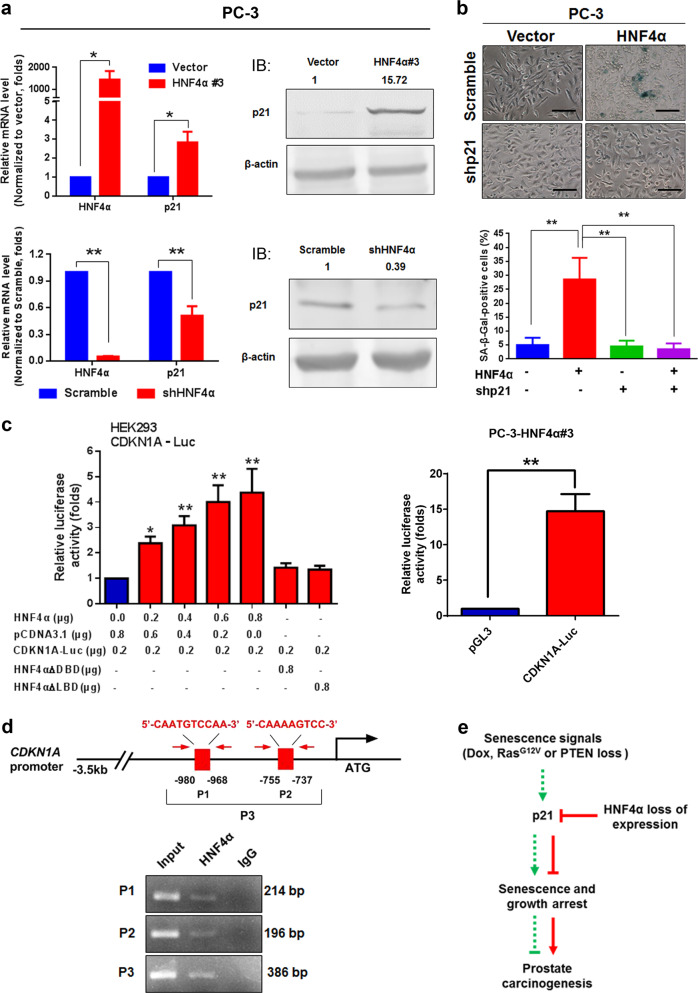
HNF4α-induced cellular senescence in prostate cancer cells is driven by its direct transactivation of p21 (*CDKN1A*) gene. **a** qRT-PCR (left) and immunoblot (right) analyses of p21 expression in HNF4α-overexpressed or HNF4α-silenced PC-3 cells. Results showed that both p21 mRNA and protein levels were significantly increased in PC-3-HNF4α infectants, but decreased in shHNF4α-PC-3 infectants. **b** Detection and scoring of SA-β-Gal-positive cells in PC-3-HNF4α infectants upon p21 knockdown. Upper: representative micrographs show the SA-β-Gal-positive cells in PC-3-HNF4α infectants with or without p21 knockdown. Lower: histogram shows the SA-β-Gal-positive cells. Results showed that knockdown of p21 could significantly reduce the number of SA-β-Gal-positive cells in PC-3-HNF4α infectants. Results were obtained from three independent experiments. Scale bars = 100 μm. **c** Luciferase reporter assay. Left: luciferase reporter assay of CDKN1A-Luc reporter performed in HEK293 cells transfected with either intact HNF4α or its truncated mutants (HNF4α-ΔDBD or HNF4α-ΔLBD). Results showed that only the intact HNF4α but not its truncated mutants could transactivate the CDKN1A-Luc reporter in a dose-dependent manner. Right: luciferase reporter assay performed in PC-3-HNF4α infectants transfected with either CDKN1A-Luc or empty reporter pGL3. Results showed that significant transactivation of CDKN1A-Luc reporter was shown in PC-3-HNF4α infectants. *P < 0.05; **P < 0.01 versus controls. **d** ChIP assay. Top: schematic diagram shows the locations of two identified HNF4α-binding sites (P1, P2) located at the proximal region of *CDKN1A* gene promoter. The sequences of HNF4α-binding sites are shown in red. The locations of ChIP-PCR primers (indicated by arrows) are also shown. Bottom: results showed that only HNF4α but not IgG-immunoprecipitated DNA fragments extracted from HNF4α-transfected HEK293 cells could be PCR-amplified at the HNF4α-binding sites. Nonimmunoprecipitated sonicated DNA (10% diluted) was used as input. **e** Schematic diagram d**e**picts the hypothesized mechanism of HNF4α-induced cellular senescence and growth arrest via its transactivation of p21 in prostate cancer cells

We next sought to determine the downstream molecular targets of HNF4α that could mediate the induction of cellular senescence in HNF4α-infectants of prostate cancer cells. It is well-characterized that activated tumor suppressors p53-p21 and p16-pRb involved pathways play significant roles in the regulation of cellular senescence in cancer cells [48]. Expression analyses showed that both mRNA and protein levels of cyclin-dependent kinase inhibitor p21^WAF1/CIP1^ (hereafter as p21) was markedly and commonly increased in all tested HNF4α infectants (AR-positive: LNCaP-HNF4α and VCaP-HNF4α; AR-negative: PC-3-HNF4α) but became significantly reduced in PC-3-shHNF4α infectants (Supplementary Figs. S6 and S7a, Fig. 8a). Increased p21 protein expression was also detected in PC-3-HNF4α xenograft tumors (Supplementary Fig. S7b). Furthermore, treatment with a senescence inducer Dox could induce concomitant increased mRNA expressions of both HNF4α and p21 in LNCaP and PC-3 cells, suggesting a direct association between two factors in prostate cancer cells (Supplementary Fig. S7c). To further verify the significance of p21 induction in the HNF4α-induced cellular senescence in PC-3-HNF4α infectants, we then silenced the p21 expression in PC-3-HNF4α infectants by shRNA and evaluated their SA-β-Gal activity status. Results showed that knockdown of p21 could significantly suppress the SA-β-Gal activity in PC-3-HNF4α infectants and restore to same levels as the empty vector-infectants (Fig. 8b). We next determined whether the induction of p21 expression in HNF4α-infectants could be a result of direct transactivation of its gene promoter by HNF4α or indirectly via its other downstream signaling molecules. Results of luciferase reporter assay indicated that only intact HNF4α but not its DBD- or LBD-truncated mutants could dose-dependently transactivate the CDKN1A-Luc reporter activity in both transfected nonprostatic HEK293 cells and prostatic PC-3-HNF4α infectants, suggesting that HNF4α was directly responsible for the induction of *CDKN1A* (p21) gene and also its transactivation would require intact HNF4α (Fig. 8c). Furthermore, ChIP analysis identified two HNF4α-binding sites (located at −980 to −737 bp) in the proximal *CDKN1* gene promoter (Fig. 8d). To elucidate whether the induction of p21 in HNF4α infectants would involve p53 or not, we examined the p53 levels in HNF4α and shHNF4α infectants. Immunoblot analysis revealed that there was no significant change in p53 protein levels in both HNF4α and shHNF4α infectants of LNCaP (expresses wild-type p53) and HNF4α infectants of DU145 (expresses mutated p53) (Supplementary Fig. S8a). Moreover, Co-immunoprecipitation (Co-IP) analysis showed that direct interaction between HNF4α and p53 was not evidenced in LNCaP cells (Supplementary Fig. S8b), suggesting that the HNF4α-mediated induction of p21 in prostate cancer cells was p53-independent. Interestingly, slight increase of *RB1* transcript level was observed in HNF4α-infectants of LNCaP but not PC-3 cells (Supplementary Fig. S7a); however, immunoblot analysis demonstrated that there was no significant change in pRb and phosphorylated pRb protein levels in HNF4α infectants of both LNCaP and PC-3 (Supplementary Fig. S8c). Moreover, results of luciferase reporter assay indicated that HNF4α could not transactivate the RB1-promoter Luc reporter activity, suggesting that the increase of RB1 mRNA level in LNCaP cells might be cell type-dependent or indirect (Supplementary Fig. S8d). Together, these results suggest that HNF4α could directly transactivate the *CDKN1* gene and induce the cellular senescence in prostate cancer cells in a p53-independent manner.

## Discussion

In the present study, we demonstrated for the first time that the orphan NR HNF4α exhibited a downregulation expression pattern in prostate cancer cell lines, prostate cancer tissues and a xenograft model of castration-relapse prostate cancer, and its downregulation was attributed to histone deacetylation and DNA methylation. The knockdown of HNF4α could significantly promote both the in vitro and in vivo malignant growth capacities (including cell proliferation, resistance to drug-induced senescence, resistance to androgen-deprivation and paclitaxel, colony formation, and in vivo tumorigenicity) of prostate cancer cells; and conversely, its overexpression could induce cellular senescence and cell-cycle arrest in prostate cancer cells. We also characterized that the HNF4α-induced inhibition of cell proliferation and cell-cycle arrest in prostate cancer cells was mediated by a mechanism of its direct transactivation of *CDKN1A* (p21^WAF1/CIP1^) gene. Previously, induction of *CDKN1A* expression is also demonstrated in F9 murine embryonal carcinoma cells and HEK293 human embryonic kidney cells upon transient HNF4α transfection [29, 49]. Our results indicate that HNF4α could perform a tumor suppressor function in cell-cycle regulation in prostate cancer cells. Indeed, the tumor suppressor function exerted by HNF4α has been demonstrated in HCC. It is shown that deletion of HNF4α can enhance cell proliferation and activate c-Myc signaling in hepatocytes, and also significantly promote the carcinogen-induced HCC progression in mice [32, 33]. Conversely, its ectopic expression can attenuate the carcinogen (diethylnitrosamine)-induced hepatic fibrosis and block HCC malignant development in a carcinogen-induced rat HCC model, which is accompanied with suppression of EMT and growth of cancer stem cells and inhibition of β-catenin activation [28].

Our present functional analysis characterized that HNF4α could perform a tumor suppressor function in prostate cancer, as evidenced by its overexpression could induce cell-cycle arrest and cellular senescence in prostatic epithelial and prostate cancer cells, and conversely its knockdown could confer resistance to either oncogene-induced or drug-induced cellular senescence in prostatic cells. In a previous study in human pancreatic β-cells, it shows that an isoform of HNF4α (HNF4α8) can act as a mitogen in β-cells to initiate cell-cycle entry but cannot sufficient for completion of cell cycle and its overexpression can induce DNA damage and cellular senescence [50]. Moreover, in a HNF4α-knockout mouse model, it shows that temporal disruption of liver-specific *Hnf4α* gene can promote proliferation of hepatocytes and affect expression of genes involved in cell cycle and cellular growth, and its loss may directly contribute to HCC development [51].

Cellular senescence, a cellular process that imposes permanent proliferative arrest on cells, is a potent mechanism of tumor suppression. Here, we demonstrated that the cell-cycle regulator and senescence-associated gene *CDKN1A* (p21^WAF1/CIP1^) was significantly induced in HNF4α-overexpressed prostate cancer cells, rather than *CDKN1B* (p27) and p53 (Supplementary Figs. S7a and S8a), indicating that HNF4α-meditated senescence in prostate cancer cells could be operated in a p53-independent manner, as evidenced by its promotion of cellular senescence in prostate cancer cells with different p53 background. Although the process of cellular senescence is shown to be regulated by p53 and or p16-pRb pathways, some studies also show that this process can be cell type-specific and regulated by different senescence pathways independent of p53 and p16 [52, 53]. Previously, we have demonstrated that another orphan NR TLX, which displays an upregulation expression in advanced prostate cancer cells, can perform an oncogenic function in prostate cancer, by suppressing cellular senescence via its differential co-regulation of *CDKN1A* and transactivation of the *SIRT1* gene in a p53-independent manner [54]. Thereby, it is speculated that this cellular process would be regulated by networks of multiple transcription factors, including NRs, in prostatic epithelial cells and cancer cells, which remains to be further elucidated.

Intriguingly, we also observed that knockdown of HNF4α could enhance the expression of AR and its variant AR45, and functionally promote the resistance to androgen-deprivation in vitro and in vivo in LNCaP prostate cancer cells (data not shown). However, it is still unclear whether the HNF4α-induced suppression of AR expression would be mediated directly by HNF4α or indirectly via other signaling. This observation also implicates that HNF4α might be involved in AR signaling in prostate cancer cells. Indeed, in a recent ChIP-sequencing study, HNF4α is identified as a tissue-specific collaborating factor for AR signaling in AR-target tissues via its constitutive binding to chromatin and through that it can guide AR to specific genomic loci upon hormone stimulation [55].

In summary, the current study provides evidence showing that the druggable NR HNF4α performs a tumor suppressor function in prostate cancer, by suppressing cellular senescence via its transactivation of C*DKN1A* (Fig. 8e). Our study also suggests that targeting of HNF4α signaling could be a potential therapeutic strategy for cellular senescence in prostate cancer.

## Materials and methods

### Immunohistochemistry

Peroxidase immunohistochemistry of HNF4α and immunoreactivity score (IRS) analysis were performed in a prostatic tissue microarray slide containing human normal prostate (*n* = 10), benign prostatic hyperplasia (BPH, *n* = 20), and prostate cancer (*n* = 50) tissues using a rabbit polyclonal anti-HNF4α antibody (HPA004712, Sigma) following procedures as described previously [56].

### Cell lines and cultures

A panel of immortalized human prostatic epithelial cell (BPH-1, RC-165N, PWR-1E), and prostate cancer cell lines (LNCaP and its metastatic C4 sublines, LAPC-4, VCaP, PC-3 and PC-3M, DU145, CA-HPV-10, ARCaP_M_) were used in this study. PWR-1E, LNCaP, LAPC-4, PC-3, and DU145 were obtained from ATCC (Manassas, VA), ARCaP_M_ from Novicure Biotechnology (Birmingham, AL) whereas other cell lines were provided by the original investigators (BPH-1 from Dr S. Hayward; C4 sublines from Dr E.T. Keller; RC-165N from Dr J.S. Rhim; VCaP from Dr K. Pienta). PWR-1E, RC-165N and CA-HPV-10 were cultured in keratinocyte serum-free medium supplemented with bovine pituitary extract and recombinant epidermal growth factor, whereas other cell lines were maintained in different media as described previously [56, 57].

### Plasmid construction

(a) Expression plasmids: full-length human HNF4α (NR2A1) was PCR-amplified using the pENTR223-HNF4α (original GenBank accession number: BC137539.1) obtained from DNASU Plasmid Repository (Biodesign Institute, Arizona State University), FLAG-tagged and subcloned into pBABE-puro (pBABE-HNF4α) for retroviral transduction, pWPI (pWPI-HNF4α) for lentiviral transduction or pcDNA3.1 (as pcDNA-HNF4α) for transfection. FLAG-tagged truncated mutants HNF4α-ΔLBD (with deletion of ligand-binding domain) and HNF4α-ΔDBD (with deletion of DNA-binding domain) were generated by a fusion-PCR method and subcloned into pcDNA3.1 for reporter gene assay. pBABE-puro-H-Ras^G12V^ was generated previously [54]. (b) Reporter plasmids: pGL3-CDKN1A-Luc with and insert of *CDKN1A* promoter sequences was constructed previously [54]. Human *RB1* gene promoter (−3221 to −84bp) were PCR-amplified from genomic DNA of LNCaP cells and cloned into pGL3 basic vector as pGL3-RB1-Luc. (c) Plasmids for RNA interference: designed DNA oligonucleotides containing the shRNA cassettes targeting HNF4α (*HNF4A*), *PTEN*, and *CDKN1A* (p21), and scramble sequences for nontargeting control were synthesized and cloned into pLKO.1-puro lentiviral vector for expressing shRNAs. pLKO.1-shPTEN was generated previously [54]. The specificities of pLKO.1-shHNF4α, pLKO.1-shPTEN, and pBABE-puro-H-Ras^G12V^ were validated in infected prostatic cells for their knockdown efficiency and expression by qRT-PCR (Supplementary Fig. S9). The sequences of oligonucleotides are listed in Supplementary Table S1. All plasmid constructs were confirmed by DNA sequencing before use.

### Viral transduction

For production of retroviruses, pBABE-FLAG-HNF4α/HNF4α-ΔDBD/ΔLBD or empty vector was transfected into PA317 packaging cells; whereas for lentivirus production, pWPI-FLAG-HNF4α, pLKO.1-puro-shHNF4α, or pLKO.1-puro-shCDKN1A (or their pLKO.1-shScrambles) was transfected into 293FT cells following procedures as described previously [58, 59]. To generate stable HNF4α-transduced prostate cancer cells, PC-3 cells were infected with pBABE-FLAG-HNF4α expressing viruses, while LNCaP cells were infected with pWPI-FLAG-HNF4α viruses followed by antibiotic selection or flow cytometry. All HNF4α-transduced cells were validated by qRT-PCR and immunoblotting before use.

### PCR and immunoblot analyses

Quantitative real-time RT-PCR (qRT-PCR) and immunoblot analyses were performed following procedures as described previously [59, 60]. Relative mRNA levels were determined using the comparative 2^−ΔΔCT^ method and normalized against β-actin. The specificity of the primers was validated by the melting-curve detection. PCR cycle times ≥ 34 were considered to be below detection. For quantitative methylation-specific PCR (qMSP), bisulfite conversion method was used (EZ DNA Methylation-Gold Kits, Zymo Research), with the type II collagen gene (*COL2A1*) used as the internal reference gene. Primer sequence information is listed in the Supplementary Table S1. For immunoblot analysis, a chemiluminescence method was used for immunosignal detection (Amersham ECL Western Blotting Detection System). Primary antibodies used are as follows: HNF4α (K9218, abcam), p21^Waf1/Cip1^ (12D1, Cell Signaling Technology), β-actin (C4, Santa Cruz Biotechnology), FLAG (M2, Sigma-Aldrich), p53 (sc-126, Santa Cruz Biotechnology), Rb (ab181616, abcam), and phospho-Rb (ab184796, abcam).

### Bioinformatics analysis

The expression profiling of HNF4α in clinical prostate cancer was analyzed using the cancer microarray datasets in the Oncomine database (http://www.oncomine.org) and Gene Expression Omnibus (GEO) database [61].

### In vitro cell growth analyses

(a) MTT assay. Subconfluent cultured cells were seeded equally at density 6 × 10^3^ cells/well in 96-well plates and cultured in growth media for 4–7 days with fresh media replaced every 3 days. Viable cells (grown for every other days) were determined by MTT assay as described previously [59]. Briefly, cells were incubated with 100 µl/well methylthiazolyldiphenyl-tetrazolium bromide (MTT, 0.5 mg/ml) in phenol red-free RPMI1640 medium for 4 h at 37°C, followed by incubation with 100 µl/well SDS-HCl solution (10% SDS, 10 mM HCl, and 5% isobutanol) overnight in a CO_2_-free incubator at 37 °C to dissolve the formed formazan dyes. Absorbance *A*_570_ was measured in a microplate spectrophotometer. (b) Invasion assay. Transwell invasion assay was performed using the 24-well Boyden chamber units (Corning, NY) with 8-µm pore-sized insert membranes precoated with Matrigel (1 mg/ml, BD Bioscience; upper surface) and fibronectin (1 mg/ml, lower surface) as described previously [62, 63]. Briefly, cells were allowed to grow to subconfluency (70–80%), serum free-starved for 12–16 h and seeded at density of 3 × 10^4^ cells/well onto the upper chamber with serum-free medium. Conditional NIH-3T3 culture medium was added to the lower chamber wells as chemoattractant. After 12–18 h incubation at 37 °C, cells migrated to the under surfaces of membranes were fixed with 4% PBS-paraformaldehyde and stained with 1% crystal violet. The stained invaded cells from five randomly captured ×200 fields were counted under a microscope. Assays were performed at least in triplicates for each experimental condition and repeated in three independent experiments. (c) Adhesion assay. Cells were starved in serum free-F12K medium for 8 h before assay, suspended in F12K with 0.1% BSA and seeded at density of 2 × 10^4^ cells/well onto collagen I-precoated 96-well plates. After brief incubation for 20 min at 37 °C, nonadherent cells were removed by gently washing the wells three times with 100 µl F12K. Adherent cells were then incubated with 200 µl F12K with 10% FBS for 4 h for recovery. Viable adherent cells were determined by MTT assay as described above. (d) Clonogenic assay. Cells were plated at density of 1 × 10^5^ cells/plate onto 100-mm plates and cultured with growth medium for 7–10 days. After brief microscopic examination for colonies formed at appropriate sizes (>50 cells), colonies were fixed with 100% methanol and stained with 0.5% crystal violet. The stained colonies were counted under a stereomicroscope at ×40.

### Cell cycle analysis

Cultured cells at 80% confluence were harvested for cell-cycle analysis following procedures as described previously [58]. DNA flow cytometry was performed on a flow cytometer (FACS Aria Fusion Flow Cytometer, BD Biosciences) and cell cycle distribution was analyzed using the ModFit LT2.0 software. Individual clones were analyzed in triplicates and repeated in three individual experiments.

### Senescence-associated β-galactosidase assay

Cytochemical staining of SA-β-Gal activity was performed on formaldehyde-fixed cultured cells following procedures as described previously [54]. After staining, the number of SA-β-Gal-positive and -negative cells were counted under a bright-field microscope in five randomly selected ×200 fields. The SA-β-Gal activity was scored by percentage of positively stained cells per total cells counted.

### Molecular biology analysis

(a) Luciferase reporter assay. Dual luciferase reporter assay was performed in prostatic PC-3 and nonprostatic HEK293 cells transfected with reporter plasmid (pGL3-CDKN1A/RB1-Luc or pGL3), expression plasmid (pcDNA3.1-HNF4α/HNF4α-ΔDBD/HNF4α-ΔLBD or pcDNA3.1) and pRL-CMV following procedures as described previously [64]. All assays were repeated in triplicates. Data were presented as mean ± SD. (b) ChIP assay. ChIP assay of *HNF4A* and *CDKN1A* promoter DNA was performed in prostatic cells or nonprostatic HEK293 cells following procedures as described previously [64]. Immunoprecipitated DNA was analyzed by PCR using primer pairs specific for human *HNF4A* and *CDKN1A* listed in the Supplementary Table S1. (c) Co-IP assay. Procedures were performed as previously described [62].

### Xenograft tumorigenicity assay

PC-3-HNF4α, PC-3-shHNF4α and their empty vector-transduced cells were evaluated of their in vivo tumorigenicity in intact male SCID mice following procedures as described previously [58]. The VCaP-CRPC tumor xenograft model of CRPC was generated as described previously [65, 66]. All animal protocols were approved by the CUHK-Animal Experimentation Ethics Committee.

### Statistical analysis

Data were expressed as mean ± SD. Differences of results were evaluated with two-tail Student’s *t* test and considered significant where *P* < 0.05.

## Supplementary information

Supplementary Table S1

Supplementary Figures and Figure Legends
