# Supplementary material for: Nuclear receptor HNF4α performs a tumor suppressor function in prostate cancer via its induction of p21-driven cellular senescence
Source: Oncogene. 2019 Nov 6;39(7):1572–89. doi: 10.1038/s41388-019-1080-3 (PMC7018660; doi:10.1038/s41388-019-1080-3)
Supplement: Supplementary file 1 — Supplementary Table S1 [file 41388_2019_1080_MOESM1_ESM.docx]

**Supplementary Table S1**

**Primers for HNF4α expression clone construction**

| HNF4α-pcDNA3.1 | Forward primer | CGCGGATCCGCGATGCGACTCTCCAAAACCCTCGT |
| --- | --- | --- |
|  | Reverse primer | CCGGAATTCCGGTTAAGCAACTTGCCCAAAGCGGC |
| HNF4α-pBABE | Forward primer | CGCGGATCCGCGATGGATTACAAGGATGACGACGATAAG |
|  | Reverse primer | CCGGAATTCCGGTTAAGCAACTTGCCCAAAGCGGCACAGT |
| HNF4α-pWPI | Forward primer | CTAGTTTAAACCACCATGGATTACAAGGATGACGACGATAAG |
|  | Reverse primer | CTAGTTTAAACCACCTTAAGCAACTTGCCCAAAGCGGCACAGT |
| HNF4α△LBD | Forward primer | GTCCCGCTCATTCTGGACGGCGACACCCAGGCTGTTGGGCG |
|  | Reverse primer | CGCCCAACAGCCTGGGTGTCGCCGTCCAGAATGAGCGGGAC |
| HNF4α△DBD | Forward primer | GATCTGCTCGATCATCTGCCAACACACATCTGCGATGCTGG |
|  | Reverse primer | CCAGCATCGCAGATGTGTGTTGGCAGATGATCGAGCAGATC |

**Primers for p21 and Rb promoter clone construction**

| pGL3-p21-Promoter | Forward primer | AAGCTCGAGGACTCATCCTAAGTGGGCAC |
| --- | --- | --- |
|  | Reverse primer | ATCAAGCTTGCGCGGCCCTGATATACAAC |
| pGL3-Rb-Promoter | Forward primer | CCTGCTAGCTCAATTGCTTCTGAGTTGTGTGG |
|  | Reverse primer | ATGAAGCTTCCTCTCCCGACTCCCGTT |

**Primers for real-time PCR**

| β-actin | Forward primer | ATGGATGATGATATCGCCGCG |
| --- | --- | --- |
|  | Reverse primer | CTCCATGTCGTCCCAGTTGGT |
| HNF4α | Forward primer | GAGCTGCAGATCGATGACAA |
|  | Reverse primer | TACTGGCGGTCGTTGATGTA |
| p27 | Forward primer | CCTGAACGGAGCTGAAGTCG |
|  | Reverse primer | CCGCTGTTTGTCTTGGAGGA |
| p16 | Forward primer | TGGGTCCCAGTCTGCAGTTA |
|  | Reverse primer | AATCCGGAGGGTCACCAAGA |
| p21 | Forward primer | AGGCAAAAGTCCTGTGTTCCA |
|  | Reverse primer | GCATGGGTTCTGACGGACAT |
| PTEN | Forward primer | CAAGATGATGTTTGAAACTATTCCAATG |
|  | Reverse primer | CCTTTAGCTGGCAGACCACAA |
| Rb | Forward primer | AGGTCTGCCAACACCAACAA |
|  | Reverse primer | TTCTTTTGAGCACACGGTCG |
| p53 | Forward primer | TTTTCCCCTCCCATGTGCTC |
|  | Reverse primer | CAATCCAGGGAAGCGTGTCA |
| Ras | Forward primer | GAGGATTCCTACAGGAAGCAAG |
|  | Reverse primer | TTGACCTGCTGTGTCGAGAA |
| COL2A1 | Forward primer | GGGAAGATGGGATAGAAGGGAATAT |
|  | Reverse primer | TCTAACAATTATAAACTCCAACCACCAA |

**Primers for ChIP PCR**

| p21 | P1 | Forward primer | TGGCATTTTTGTCATTTTGGAG |
| --- | --- | --- | --- |
|  |  | Reverse primer | TATGCCCGATACACCCCTCATAAG |
|  | P2 | Forward primer | CAGACAACTCACTCGTCAAATCCT |
|  |  | Reverse primer | TGATTGTCACATGCTTCCG |
|  | P3 | Forward primer | TGGCATTTTTGTCATTTTGGAG |
|  |  | Reverse primer | TGATTGTCACATGCTTCCG |
| H3AC |  | Forward primer | ATGGAGACAGCAACAGTCCC |
|  |  | Reverse primer | ACCGCCCGGTTATCTTATTGA |

**Primers for HNF4α-shRNA clone construction**

| HNF4α-shRNA1 | Forward primer | CCGGTGCAGATGTGTGTGAGTCCATTCAAGAGATGGACTCACACACATCTGCTTTTTGGAAAC |
| --- | --- | --- |
|  | Reverse primer | AATTCGTTTCCAAAAAGCAGATGTGTGTGAGTCCATCTCTTGAATGGACTCACACACATCTGC |
| HNF4α-shRNA2 | Forward primer | CCGGTCCACATGTACTCCTGCAGATTCAAGAGATCAGCAGGAGTACATGTGGTTTTTGGAAAC |
|  | Reverse primer | AATTCGTTTCCAAAAACCACATGTACTCCTGCTGATCTCTTGA-ATCTGCAGGAGTACATGTGG |
| HNF4α-shRNA3 | Forward primer | CCGGTCGAGCAGATCCAGTTCATCAATCAAGAGTTGATGAACTGGATCTGCTCGTTTTTGGAAAC |
|  | Reverse primer | AATTCGTTTCCAAAAACGAGCAGATCCAGTTCATCAACTCTTGATTGATGAACTGGATCTGCTCG |

**Primers for p21-shRNA clone construction**

| p21-shRNA 1 | Forward primer | CCGGTGAGCGATGGAACTTCGACTTTTCAAGAGAAAGTCGAAGTTCCATCGCTCTTTTTGGAAAC |
| --- | --- | --- |
|  | Reverse primer | AATTGTTTCCAAAAA-GAGCGATGGAACTTCGACTTTCTCTTGAAAAAGTCGAAGTTCCATCGCTC |
| p21-shRNA 2 | Forward primer | CCGGTCGCTCTACATCTTCTGCCTTATCAAGAGTAAGGCAGAAGATGTAGAGCGTTTTTGGAAAC |
|  | Reverse primer | AATTGTTTCCAAAAACGCTCTACATCTTCTGCCTTACTCTTGATAAGGCAGAAGATGTAGAGCG |
| p21-shRNA 3 | Forward primer | CCGGTGACCATGTGGACCTGTCACTGTCAAGAG-CAGTGACAGGTCCACATGGTCTTTTTGGAAAC |
|  | Reverse primer | AATTGTTTCCAAAAAGACCATGTGGACCTGTCACTGCTCTTGACAGTGACAGGTCCACATGGTC |

**Primers for quantitative methylation-specific PCR (qMSP) within the promoter of HNF4α**

| Forward primer | TGAGTTAAGGGTTAAATGAGTGTTC |
| --- | --- |
| Reverse primer | TATTAATTTCTAACTAACACCCGAA |
