# Supplementary material for: Nuclear receptor HNF4α performs a tumor suppressor function in prostate cancer via its induction of p21-driven cellular senescence
Source: Oncogene. 2019 Nov 6;39(7):1572–89. doi: 10.1038/s41388-019-1080-3 (PMC7018660; doi:10.1038/s41388-019-1080-3)
Supplement: Supplementary file 2 — Supplementary Figures and Figure Legends [file 41388_2019_1080_MOESM2_ESM.pdf]

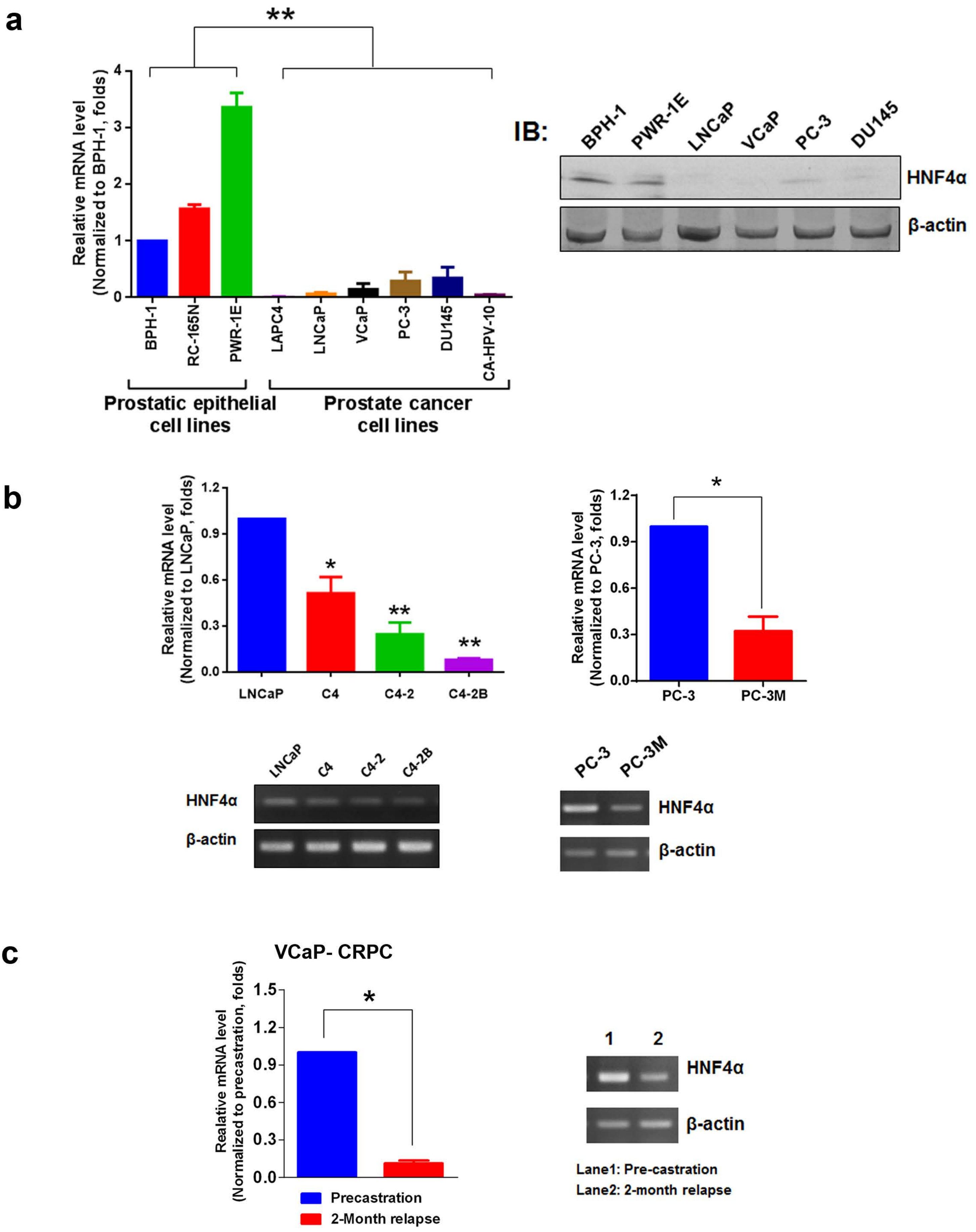

Supplementary Figure S1

Supplementary Fig. S1. HNF4α exhibited a significant decreased expression in prostate cancer. **a** qRT-PCR and immunoblot analyses of HNF4α in immortalized prostatic epithelial and prostate cancer cell lines. Significant decreases of HNF4α transcripts and protein levels were detected in a panel of prostate cancer cell lines as compared to immortalized nonmalignant prostatic epithelial cell lines. **b** Similar decreases of HNF4α transcripts were also detected in various metastatic sublines of LNCaP (C4, C4-2 and C4-2B) and PC-3 (PC-3M) as compared to their parental lines. *, $P < 0.05$, **, $P < 0.01$ versus immortalized or parental lines. **c** qRT-PCR analysis of HNF4α in VCaP-CRPC xenograft model. Result showed that a significant decrease of HNF4α transcripts was shown in castration-relapse xenograft tumors grown in castrated mice as compared to pre-castration tumors grown in intact hosts. *, $P < 0.05$ versus pre-castration tumors.

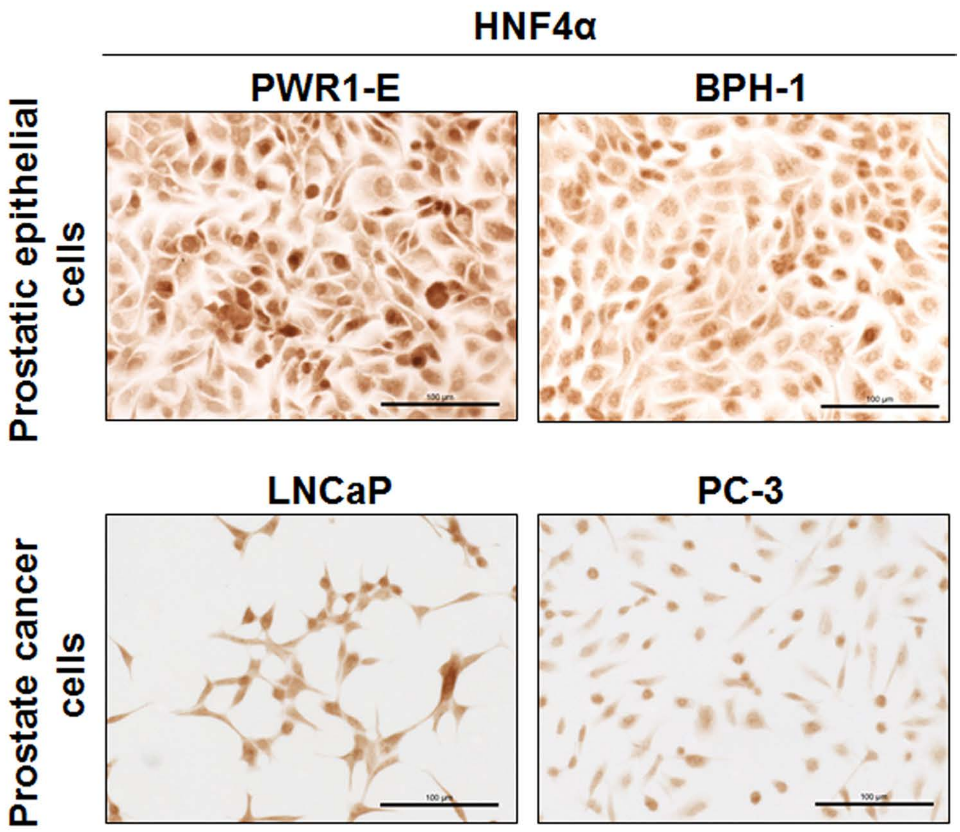

Supplementary Figure S2

Supplementary Fig. S2. Immunohistochemistry revealed that HNF4α showed a lower nuclear expression in prostate cancer cell lines (LNCaP and PC-3) as compare to immortalized epithelial cell lines (PWR-1E and BPH-1). Scale bars = 100 μm.

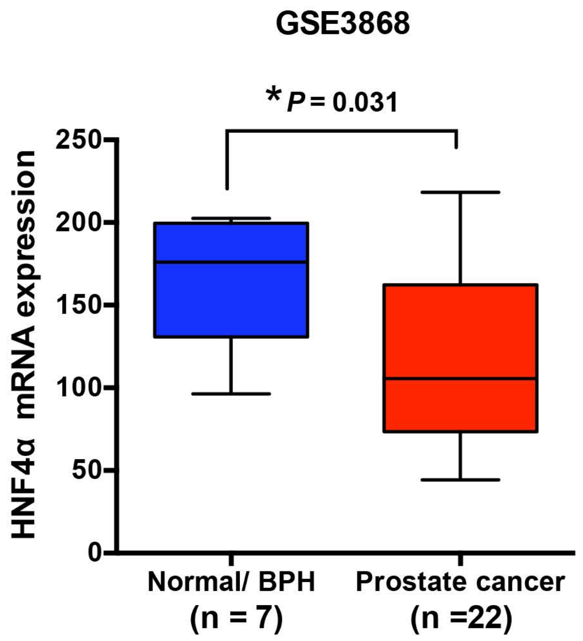

Supplementary Figure S3

Supplementary Fig. S3. Expression profile of HNF4α as revealed from a GEO dataset (GSE3868). Analysis showed that HNF4α mRNA levels exhibited a significant decrease in prostate cancer samples as compared to normal prostate gland/BPH. *, $P < 0.05$

a

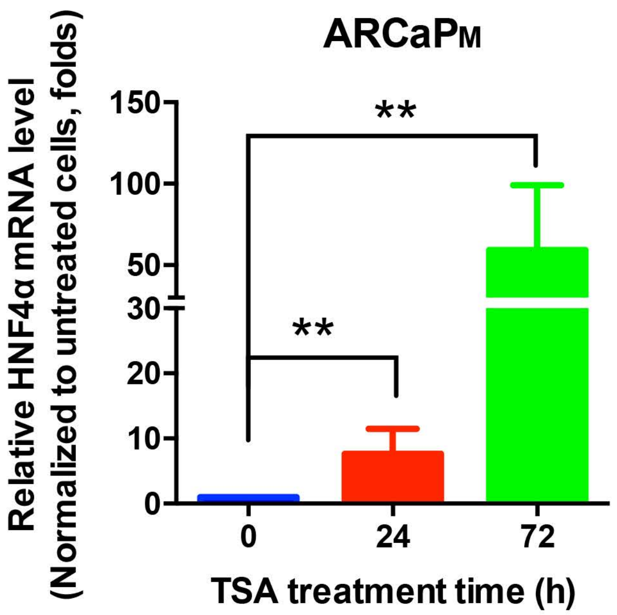

b

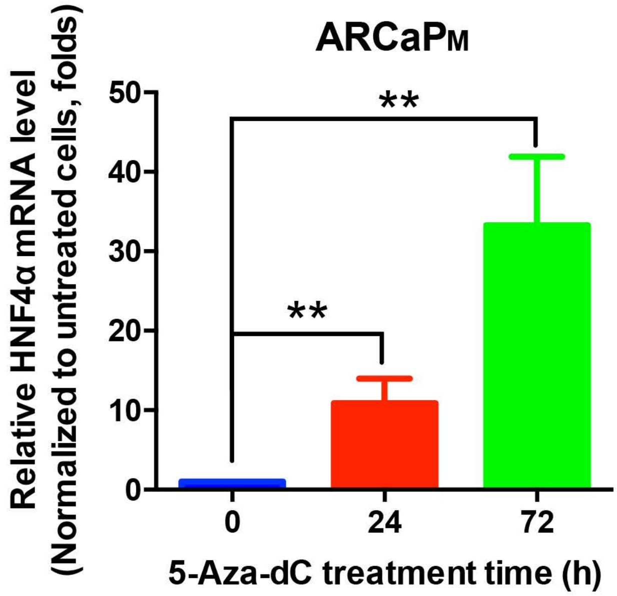

Supplementary Figure S4

Supplementary Fig. S4. Inhibition of histone deacetylase and DNA methyltransferase enhanced HNF4α expression in $ARCaP_M$ cells. **a, b** qRT-PCR analysis of HNF4α mRNA expression in $ARCaP_M$ cells upon treatments with inhibitors of histone deacetylase (TSA, 200 ng/ml) and DNA methyltransferase (5-aza-dC, 100 nM) for 24-72 h. Results showed that treatments with either TSA or 5-aza-dC could significantly elevate the HNF4α expression in a time-dependent manner. Relative HNF4α mRNA levels were normalized to β-actin. **, $P <$ 0.01 versus untreated control.

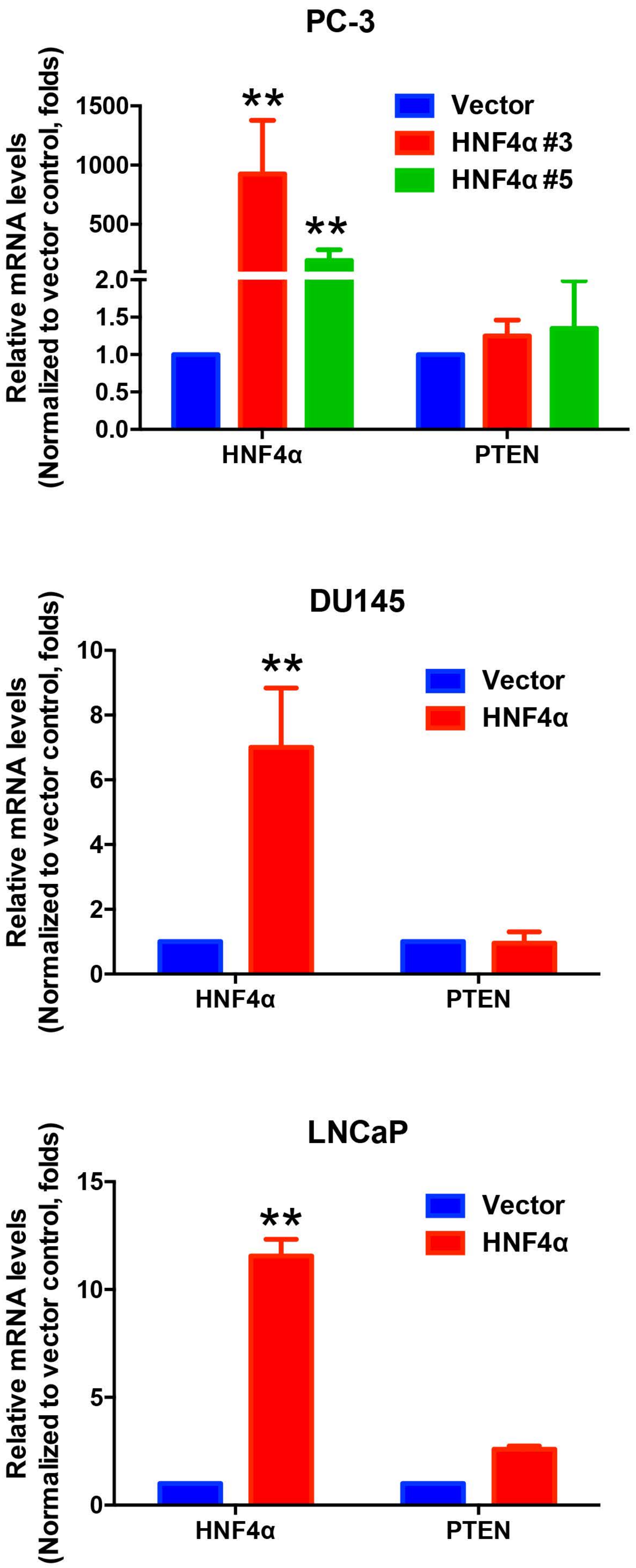

Supplementary Figure S5

Supplementary Fig. S5. qRT-PCR showed that ectopic HNF4α overexpression induced no significant changes in *PTEN* transcript levels in prostate cancer cells. **, $P < 0.01$ versus vector control.

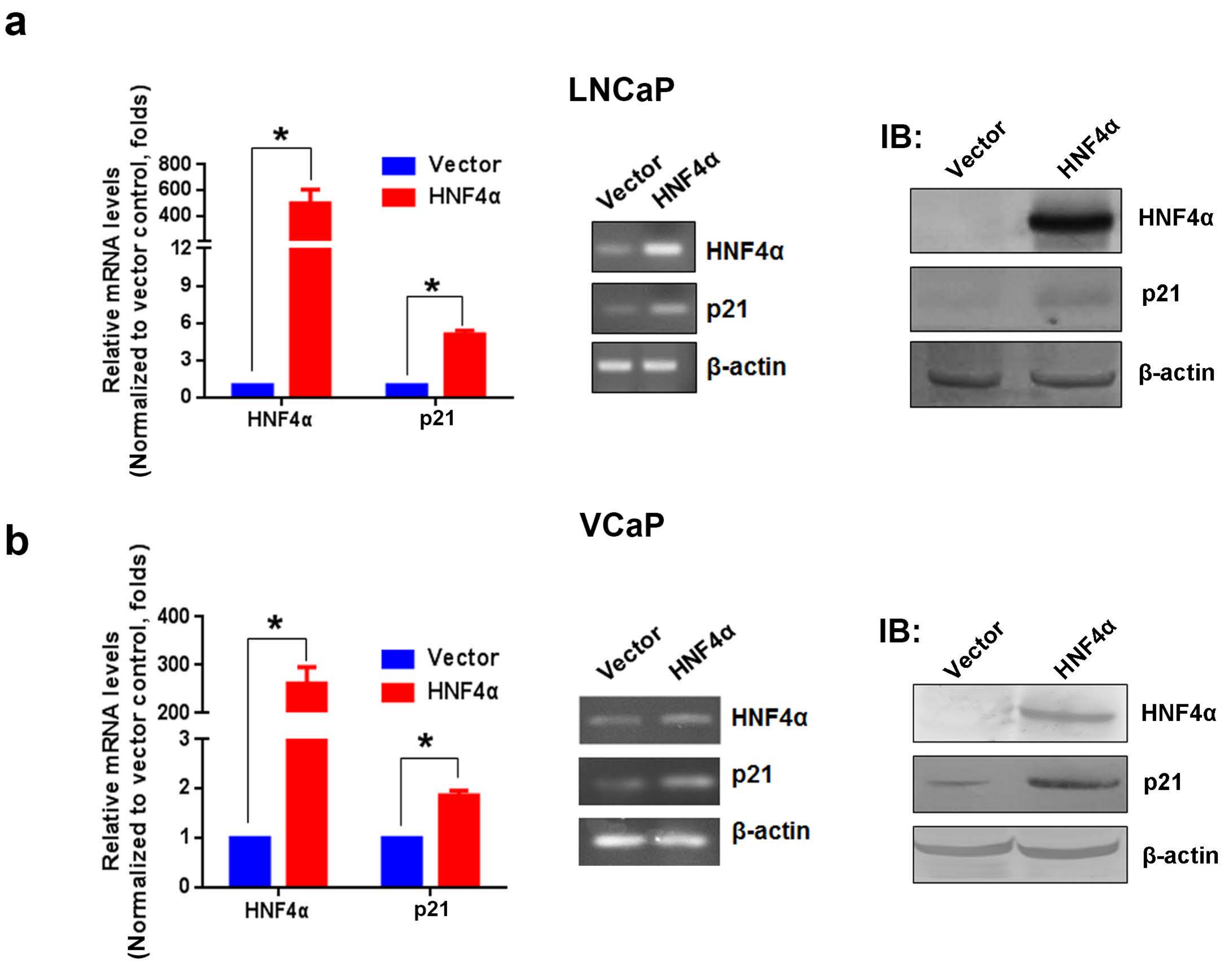

Supplementary Figure S6

Supplementary Fig. S6. p21 was significantly upregulated in HNF4α-overexpressed LNCaP **(a)** and VCaP **(b)** cells. Left: qRT-PCR analysis; Middle: representative images of agarose electrophoresis of corresponding PCR products; Right: immunoblotting of HNF4α and p21. *, $P < 0.05$ versus vector control.

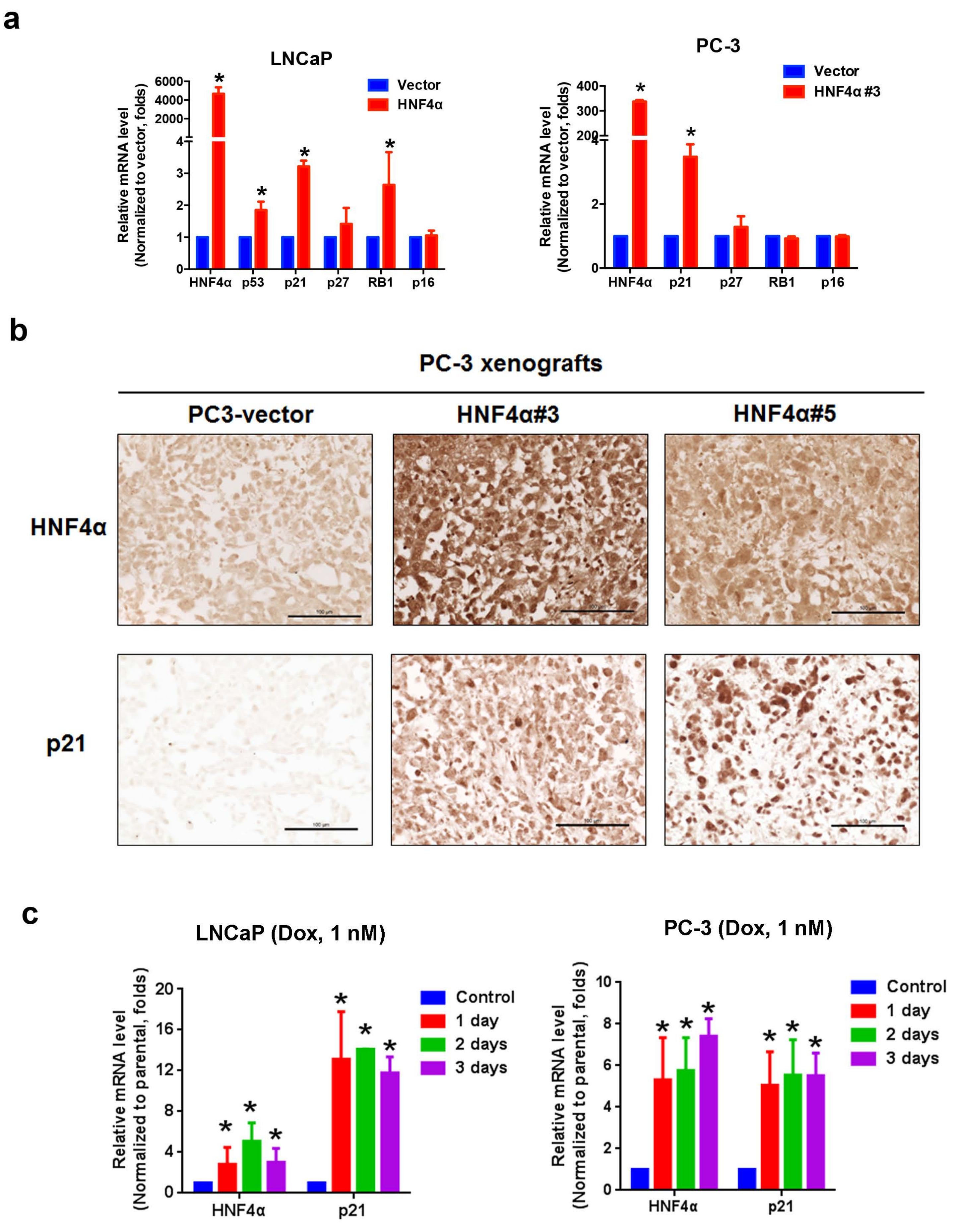

Supplementary Figure S7

Supplementary Fig. S7. Induction of p21 expression by HNF4α overexpression in prostate cancer cells. **a** qRT-PCR analysis of multiple cell cycle regulators (p53, p21, p27, pRb and p16) in LNCaP-HNF4α and PC-3-HNF4α infectants. Results showed that common induction of p21 was detected in both AR-positive LNCaP-HNF4α and AR-negative PC-3-HNF4α infectants, as compared to their vector-infectants. **b** Immunohistochemistry analysis of HNF4α and p21. The tumor cells of PC-3-HNF4α xenograft tumors expressed intense p21 immunosignal, whereas the tumor cells of PC-3-vector tumors expressed no or barely detectable p21 immunosignal. Scale bars = 100 μm. **c** qRT-PCR analysis of HNF4α and p21 in Dox-treated LNCaP and PC-3 cells. Upon short-duration (1-3 days) treatment with Dox (1 nM), significant increases of both HNF4α and p21 transcripts were induced in both LNCaP and PC-3 cells. *, $P < 0.05$ versus control group.

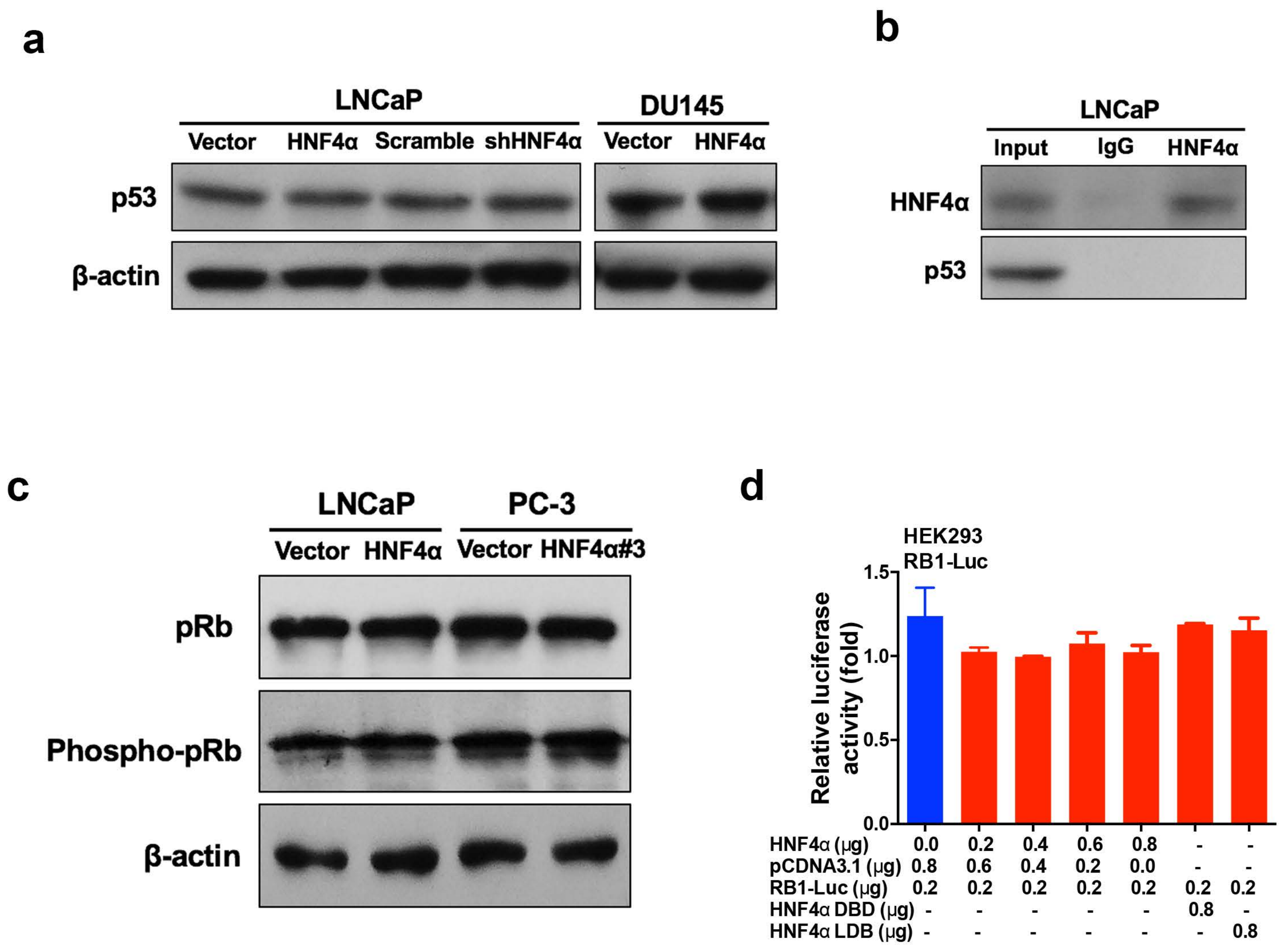

Supplementary Figure S8

Supplementary Fig. S8. a Immunoblot analysis of p53 in HNF4α and shHNF4α infectants; No significant change in p53 levels was detected in HNF4α and shHNF4α infectants of LNCaP, and HNF4α infectants of DU145 cells. **b** Co-IP assay performed in LNCaP cells. Protein lysates were immunoprecipitated with HNF4α or IgG antibody, followed by immunoblotting of HNF4α or p53. **c** Immunoblot analysis of pRb and phosphorylated pRb in HNF4α infectants. No significant change in pRb and phosphorylated pRb levels was detected in HNF4α infectants of LNCaP and PC-3 cells. **d** Luciferase reporter assay. Results indicated that HNF4α could not significantly transactivate the RB1-Luc reporter activity.

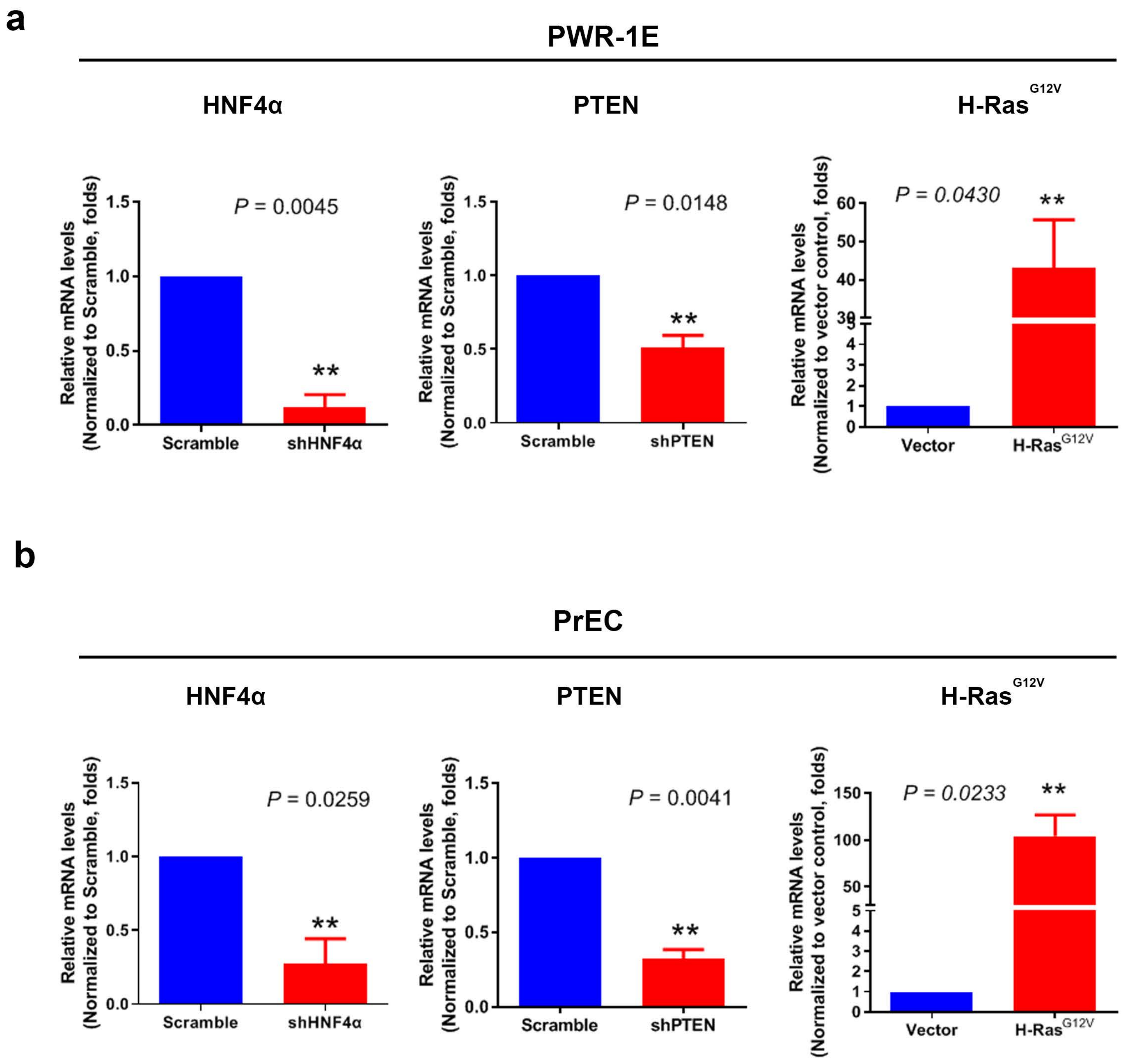

Supplementary Figure S9

Supplementary Fig. S9. Validation of shRNA-knockdown of HNF4α or PTEN, and ectopic expression of activated oncogene H-Ras^{G12V} in (**a**) PWR-1E immortalized prostatic epithelial cells and (**b**) PrEC primary cultured prostatic epithelial cells as assayed by qRT-PCR. **, $P < 0.01$ versus vector control.
